# Management of Vancouver B3 Periprosthetic Femur Fracture Using a Modular Uncemented Long Femoral Stem Prosthesis With Cerclage Wiring Technique

**DOI:** 10.7759/cureus.53895

**Published:** 2024-02-09

**Authors:** Anmol Suneja, Hitendra S Wamborikar, Sanjay V Deshpande, Vivek H Jadawala, Salahuddin Ahmed, Sachin Goel

**Affiliations:** 1 Department of Orthopaedics, Jawaharlal Nehru Medical College, Datta Meghe Institute of Higher Education and Research, Wardha, IND

**Keywords:** total hip arthroplasty, cerclage wiring, uncemented, modular, long stem prosthesis, vancouver classification, periprosthetic femur fracture

## Abstract

Periprosthetic fractures (PPF) of the femur in connection with total hip arthroplasty are becoming common and also frequently challenging to repair. Such patients typically are frail, elderly, and have osteoporosis. Owing to a scarcity of research there are no clear strategies for its effective management. However, the Vancouver classification may help in facilitating treatment decisions. For fractures around a loose femoral prosthesis (types B2 and B3), revision using a modular uncemented long stem, with or without additional fracture fixation, has been known to provide a reliable outcome. It is prudent to treat osteoporosis for fracture healing and to prevent further fractures. In this case report, we share our experience with the use of an uncemented modular long femoral stem prosthesis with a cerclage wiring technique for the management of Vancouver type B3 PPF of the left femur in a 63-year-old male patient. Revision arthroplasty using a long stem prosthesis with a cerclage wiring technique can provide better fixation, stability, and functional outcomes for the patient.

## Introduction

In connection with total hip arthroplasty, periprosthetic fractures (PPF) of the femur are becoming common and frequently challenging to repair [1]. The incidence of periprosthetic femur fracture, first noted in 1954, is currently 4.1% [2], and the rates for uncemented and revision total hip arthroplasty have increased [3]. It can occur due to a number of established risk factors including female gender, rheumatoid arthritis, having significant osteolytic lesions (particularly in high-stress anatomical regions in young patients having high activity levels, or in older populations, owing to osteoporosis) [4]. Osteoporosis must be treated to promote fracture healing and stop additional fractures [1].

Femoral periprosthetic fractures can develop during or after surgery, and their management is challenging. According to evidence, nonoperative therapy modalities like traction have disappointing outcomes [1]. Duncan and Masri devised the Vancouver classification, which offers a useful evaluation of postoperative femoral fractures based on the degree of fracture and the presence of either a loose or well-fixed component [5]. This classification also facilitates the decisions for treatment [6].

Most evidence points to stabilization of the fracture surgically using plates, strut grafts, or a combination of the two in cases of Vancouver B1 (stable prosthesis) and Vancouver C (fractures well below the implant) [1]. According to statistics, loose stems (Vancouver B2 and B3) account for over 75% of PPF [7]. Their most widely accepted treatment has been found to involve revision with a substantially porous-coated uncemented long stem, which may further involve fracture fixation with allografts and cerclage wiring in cases of Vancouver B3 fractures [8]. When an elderly patient has a cement mantle that is securely attached, cement-in-cement correction with a long-stem prosthesis is possible.

In this case report, we are reporting the case of a 63-year-old treated for Vancouver type B3 PPF of the left femur using a modular long stem, uncemented cerclage technique.

## Case presentation

A 63-year-old male presented to our hospital with chief complaints of pain and inability to bear weight on his left thigh for 15 days. The patient gave an alleged history of slipping and had a fall in his house 15 days ago, sustaining an injury to his left thigh. He immediately developed pain and swelling over the left hip and thigh region, which were gradually increasing. The pain got aggravated while moving the limb and decreased when taking rest. The patient has been unable to bear weight on his left lower limb since then.

**Figure 1:**
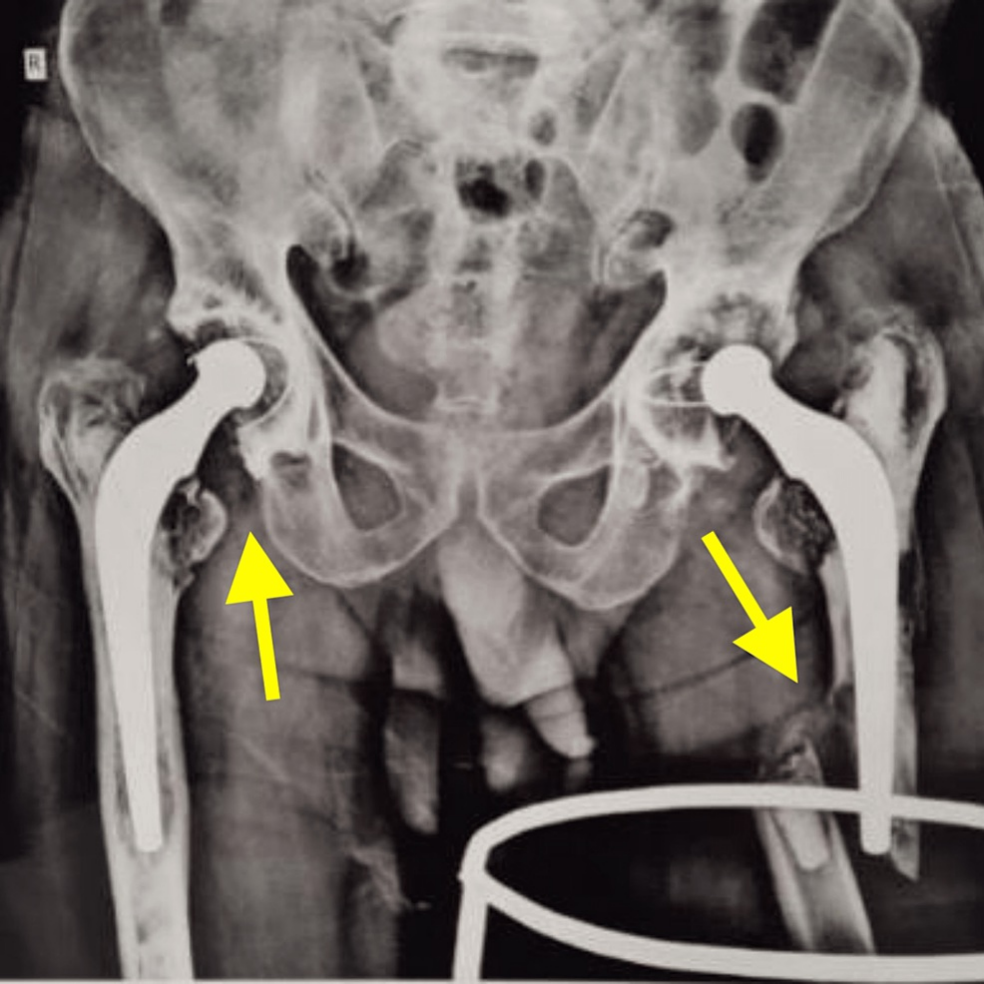
Pre-operative radiograph of pelvis with both hip joints (PBH) anteroposterior view shows bilateral total hip arthroplasty with periprosthetic femur fracture left side

**Figure 2:**
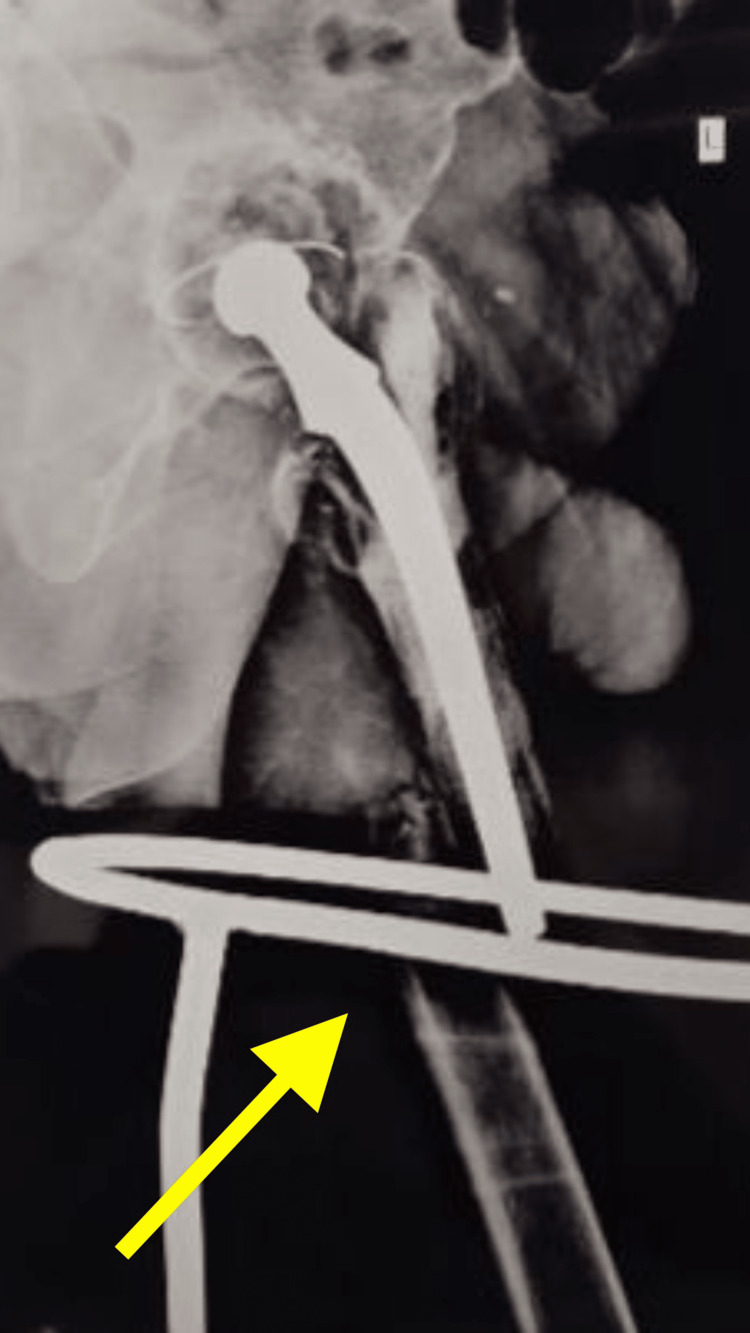
Pre-operative radiograph of the left hip with thigh lateral view showing periprosthetic femur fracture left side

**Figure 3:**
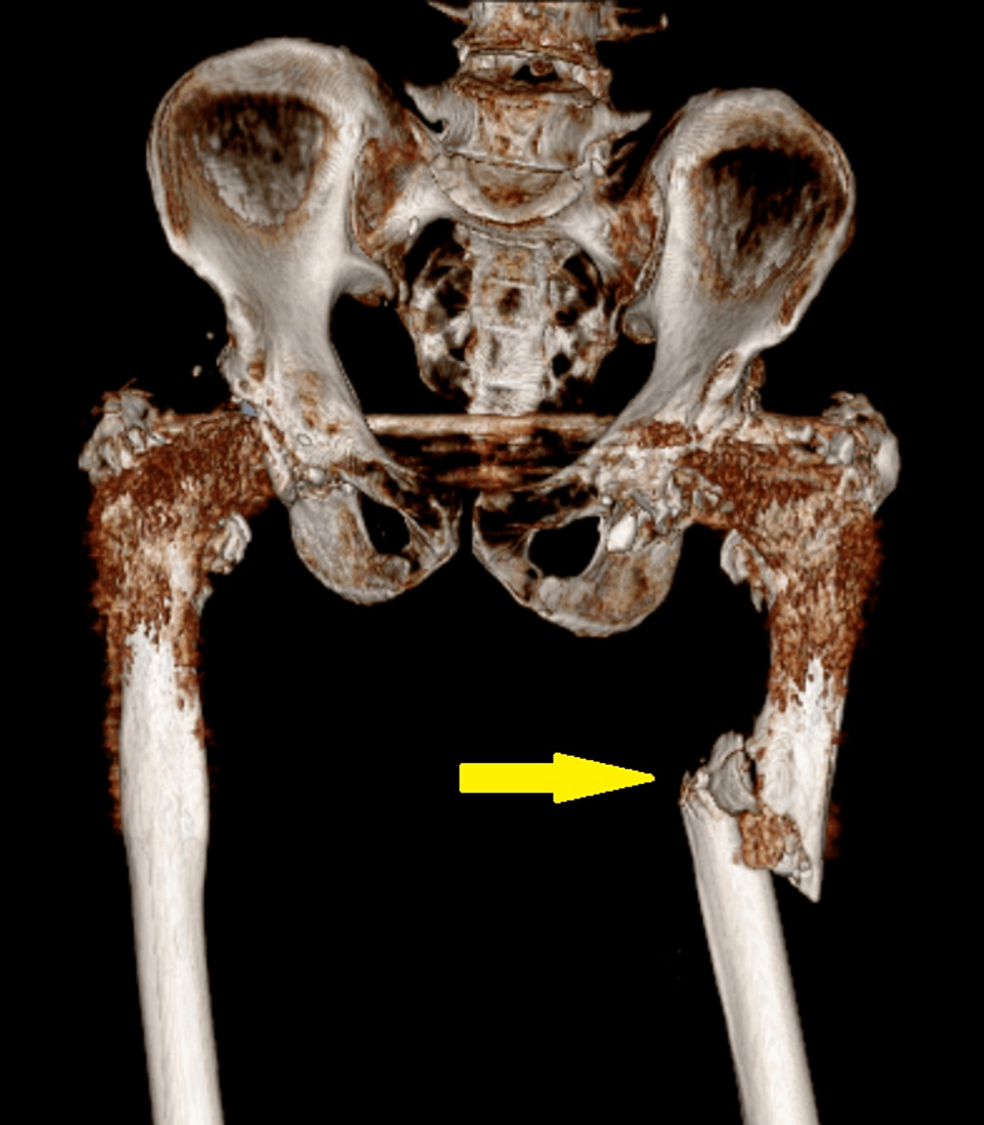
Pre-operative 3D reconstruction computed tomography scan of pelvis with both hip joints shows periprosthetic femur fracture left side

The patient also had a history of arthritis of the bilateral hip joint secondary to avascular necrosis of the bilateral femoral head for the last 15 years, which was managed with bilateral total hip replacement. X-rays and CT scans of the pelvis including both hips were done. The patient was diagnosed with a periprosthetic fracture over the shaft of the left femur in an operated case of cemented total hip replacement on the left side (Figures 1, 2, 3).

The patient's general examination was unremarkable. On physical examination of the left thigh, there was a healed scar mark from previous surgery. Bony tenderness was present over the middle 1/3rd of the femur shaft. The range of movement in the hip and knee could not be elicited due to pain. There was no distal neuro-vascular deficit. Therefore, he was then planned for revision total hip arthroplasty of the left side. 

**Figure 4:**
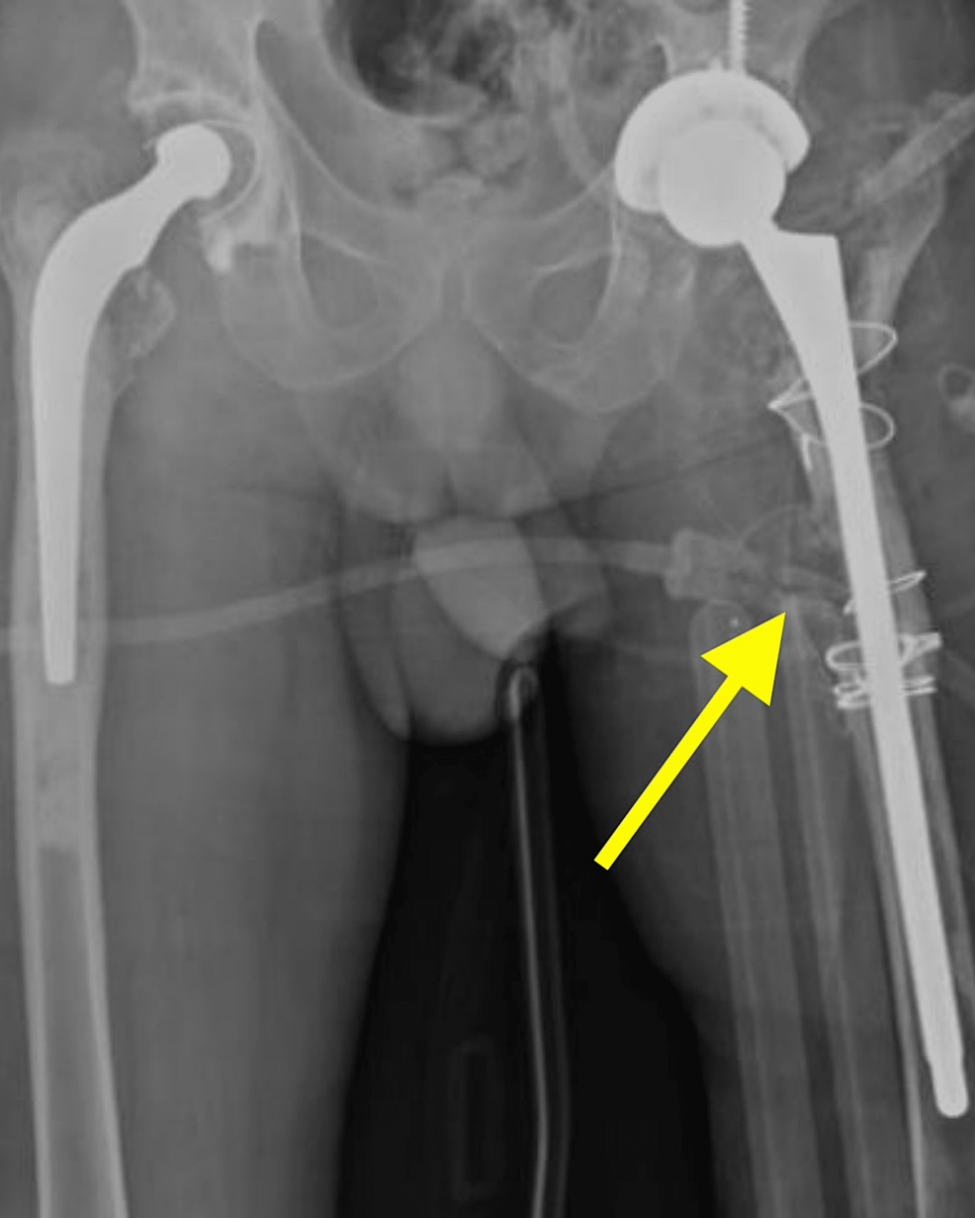
Post-operative radiograph of pelvis with both hip joints anteroposterior view shows periprosthetic femur fracture managed with revision total hip arthroplasty left side

A postero-lateral approach to the left hip was used, and about 20 cm incision was made over the postero-lateral aspect of the left hip over the previous incision scar. Skin, superficial fascia, and deep fascia were dissected. Tensor fascia lata was cut, external rotators were exposed, and it was found to be fibrosed. Fibrosed external rotators were tagged and cut with the implant being exposed. The greater trochanter and lesser trochanter were found to be fractured. The femoral stem was removed, and cement removal was done with the help of osteotome and curette. A trial of the femoral stem was taken. The acetabular cup and the liner were removed, and the acetabulum was completely exposed. Reaming was done. The acetabular cavity was prepared with serial reamers up to 56 and found to be protrusio. Bone graft harvested from the acetabulum reaming was used and inserted at the center of the acetabulum. A cup size 56 was used, and the liner was inserted. Two Screws of lengths 20 mm and 30 mm were inserted through the cup. A trial of an uncemented stem of size 12 was done, and reduction was achieved with a standard-sized trial head, which was found to be satisfactory. Five encirclages by SS wire were done near the fracture site to hold the reduction at the greater trochanter, lesser trochanter, and peri-prosthetic fracture sites. An uncemented stem of size 12 was inserted. A standard head of +5 was placed, and a reduction of the head into the acetabular cup was made. The reduction was found satisfactory and checked in flexion, abduction, adduction, and internal and external rotation. A thorough wash was given with normal saline. The drain was placed in situ. Closure is done in layers. Sterile dressing was done. A post-operative X-ray of the pelvis with both hip joints was taken and was found to be satisfactory (Figure 4).

Post-operatively, the patient was allowed full weight-bearing mobilization from day two onwards, along with static and dynamic quadriceps and hamstring strengthening exercises. Ankle pumps, knee range of movements, and hip range were also initiated from day two onwards as per pain tolerance. Hip range of movements, flexion 0 to 100 degrees and extension 0 to 10 degrees, abduction 0 to 30 degrees, and adduction 0 to 5 degrees, were achieved by the end of the second week post-operatively. Post-operatively no signs of infection or complications were seen and an X-ray one year following surgery showed good signs of fracture healing.

## Discussion

The objectives of treatment for PPF of the femur involve the union of the fracture in almost anatomical alignment, along with a sturdy prosthesis, rapid mobility with the restoration of pre-fracture function, and a reasonable prosthesis survival rate [9]. Due to risk factors including osteoporosis, low bone mass, reduced mobility, and frequently the presence of co-morbidities, it could not always be simple to achieve all these goals in patients [10]. Cerclage cables, bone grafts, and long stems were preferred over screw-plate fixation for Vancouver B2 and B3 or traction for mid- and distal-stem fractures.

The Vancouver types B2 and B3 are considered difficult periprosthetic femoral fractures to treat. As stated in a paper by Lindahl et al., open reductions and internal fixation are options only for Vancouver B1 (and C) fractures, whereas Vancouver B2 and B3 are mostly treated with revision using long-stem prosthesis [11]. However, because the femoral stem is loose, Duncan and Masri claim that Vancouver types B2 and B3 fractures can be treated with revision with a long stem, whereas in cases of Vancouver B3 fractures, allografts and cerclage wiring can be utilized [5].

According to recent literature, long femoral stems are recommended to manage PPF of Vancouver categories B2 and B3. Long femoral stem prosthesis functions like an intramedullary nail locking at the distal attachment, enabling simultaneous revision arthroplasty and fracture repair. Additionally, the fixation point is away from the fracture; subsequently, the fracture has no bearing on the stability of the stem, and the undesired effects of the fixation have no bearing on the fracture's ability to heal [12]. Modular stems are also advantageous over non-modular ones when controlling leg length inequality [10].

The available data also prefers uncemented and modular stems fixed distally over cemented techniques for repeat surgery in the B2 and B3 cases. They involve a less complex surgical procedure, providing stable diaphyseal fixation and limiting version change. In cases where the proximal metaphysis has been injured, these stems might also offer trustworthy osteo-integration [13].

Cement may leak into the fracture site when cemented procedures prevent fracture healing [14]. The cemented approach also appears to have a higher rate of nonunion and early loosening [15]. Additionally, compared to uncemented prostheses, which had a 7% refracture rate, cemented prostheses had a 15% greater refracture rate [16]. Numerous more investigations have confirmed that extensive-coated, uncemented, long-stem prostheses are superior to partial-coated or cemented ones [17]. Henceforth, it was decided to treat the B2 and B3 categories of PFFs without cement to prevent the negative consequences of cement.

Lastly, individuals having co-morbidities or possible risk factors might also experience difficulties and poor outcomes; therefore, identifying these risk factors before surgery can help surgeons anticipate potential complications and the prognosis for their patients [10]. Even though types B2 and B3 femoral periprosthetic fractures are amongst the more challenging operations for reconstruction, non-cemented distally locked long femoral stem with or without cortical on lay strut allografts provided a helpful and effective management strategy.

## Conclusions

For management of Vancouver categories B2 and B3 periprosthetic fractures (PPFs) after total hip arthroplasty with modular, long-femoral stem prosthesis that are not cemented, either with or without cortical strut allograft or cerclage wiring is beneficial in promoting fracture healing and even improves the patient’s quality of life. The B2 and B3 categories of PPFs should be treated routinely with this prosthesis revision surgery.

## References

[1] Marsland D, Mears SC (2012). A review of periprosthetic femoral fractures associated with total hip arthroplasty. Geriatr Orthop Surg Rehabil.

[2] Horwitz IB, Lenobel MI (1954). Artificial hip prosthesis in acute and nonunion fractures of the femoral neck: follow-up study of seventy cases. J Am Med Assoc.

[3] Berry DJ (1999). Epidemiology: hip and knee. Orthop Clin North Am.

[4] Berry DJ (2003). Periprosthetic fractures associated with osteolysis: a problem on the rise. J Arthroplasty.

[5] Duncan CP, Masri BA (1995). Fractures of the femur after hip replacement. Instr Course Lect.

[6] Brady OH, Kerry R, Masri BA, Garbuz DS, Duncan CP (1999). The Vancouver classification of periprosthetic fractures of the hip: a rational approach to treatment. Tech Orthop.

[7] Lindahl H, Malchau H, Herberts P, Garellick G (2005). Periprosthetic femoral fractures: classification and demographics of 1049 periprosthetic femoral fractures from the Swedish National Hip Arthroplasty Register. J Arthroplasty.

[8] Rayan F, Konan S, Haddad FS (2010). Uncemented revision hip arthroplasty in B2 and B3 periprosthetic femoral fractures - a prospective analysis. Hip Int.

[9] Masri BA, Meek RM, Duncan CP (2004). Periprosthetic fractures evaluation and treatment. Clin Orthop Relat Res.

[10] Canbora K, Kose O, Polat A, Aykanat F, Gorgec M (2013). Management of Vancouver type B2 and B3 femoral periprosthetic fractures using an uncemented extensively porous-coated long femoral stem prosthesis. Eur J Orthop Surg Traumatol.

[11] Lindahl H, Garellick G, Regnér H, Herberts P, Malchau H (2006). Three hundred and twenty-one periprosthetic femoral fractures. J Bone Joint Surg Am.

[12] Fink B, Grossmann A, Singer J (2012). Hip revision arthroplasty in periprosthetic fractures of vancouver type B2 and B3. J Orthop Trauma.

[13] Sporer SM, Paprosky WG (2004). Femoral fixation in the face of considerable bone loss: the use of modular stems. Clin Orthop Relat Res.

[14] Zaki SH, Sadiq S, Purbach B, Wroblewski BM (2007). Periprosthetic femoral fractures treated with a modular distally cemented stem. J Orthop Surg (Hong Kong).

[15] Mont MA, Maar DC (1994). Fractures of the ipsilateral femur after hip arthroplasty. J Arthroplast.

[16] Beals RK, Tower SS (1996). Periprosthetic fractures of the femur: an analysis of 93 fractures. Clin Orthop Relat Res.

[17] Springer BD, Berry DJ, Lewallen DG (2003). Treatment of periprosthetic femoral fractures following total hip arthroplasty with femoral component revision. J Bone Joint Surg Am.

